# Amniotic band syndrome

**DOI:** 10.11604/pamj.2020.36.86.20345

**Published:** 2020-06-12

**Authors:** Tsakiridis Ioannis, Dagklis Themistoklis

**Affiliations:** 1 Third Department of Obstetrics and Gynaecology, Faculty of Medicine, Aristotle University of Thessaloniki, Thessaloniki, Greece

**Keywords:** Amniotic band syndrome, primiparous, gestation

## Image in medicine

A 30-year-old primiparous woman underwent routine nuchal translucency scan at 12+1 weeks of gestation. A possible amniotic band with entrapment of the upper limbs and the umbilical cord was noted. Amniotic bands or constriction rings may obstruct vascular and lymph supply and cause deformity, amputation or even intrauterine death. The exact etiology is unknown but they may be the result of rupture of the amnion without rupture of the chorion. In some cases, a fetoscopic intrauterine procedure may release the entangled limb, thus avoiding amputation. In this case, a follow up scan was scheduled at 14 gestational weeks to reassess and plan possible treatment, however, at that time a missed miscarriage was diagnosed, probably due to constriction of the umbilical cord. Following medical management, the abortus showed an amniotic band that caused amputation of both hands and constriction of the umbilical cord, as sonographically suspected.

**Figure 1:**
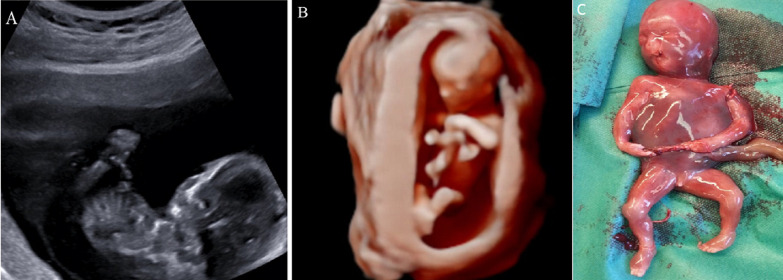
amniotic band syndrome (A, B, C)

